# Maximising relational capabilities and minimising restrictive practices in acute mental health units: the Safe Steps for De-escalation evaluation

**DOI:** 10.3389/fpsyt.2025.1676743

**Published:** 2025-11-06

**Authors:** Esario I V Daguman, Jacqui Yoxall, Richard Lakeman, Marie Hutchinson

**Affiliations:** 1 Faculty of Health, Southern Cross University, Coffs Harbour, NSW, Australia; 2 Faculty of Health, Southern Cross University, Lismore, NSW, Australia; 3 School of Nursing and Midwifery, University of Southern Queensland, Toowoomba, QLD, Australia

**Keywords:** de-escalation, psychiatric nursing, mental health services, coercion, psychiatric hospital, intervention evaluation

## Abstract

**Objectives:**

De-escalation is widely endorsed as an intentional strategy to replace and reduce restrictive practices in acute mental health units. However, high-quality evidence for its effective implementation remains limited. In response, a pragmatic, complexity-informed evaluation was undertaken to generate empirical support for the impact of an intervention, *Safe Steps for De-escalation*, on restrictive practices. The intervention centres on a four-step framework for therapeutic responding, with implementation supported by co-designed training and restrictive practice reviews.

**Methods:**

A mixed concurrent control study was conducted in three adult inpatient units in New South Wales, Australia, from March 2023 to April 2025. *A priori* weighted linear, linear mixed-effects, and generalised linear mixed-effects models were fitted between and within groups, to assess the impact of the intervention on restrictive practice events, including seclusion, physical restraint, as-needed intramuscular psychotropics, event duration, and physical injury. *A priori* hierarchical cluster analysis and between-cluster comparison were used to examine the most active de-escalation response components and any associated concurrent supplementary strategies contributing to the overall impact.

**Results:**

Compared to three control sites, implementation sites had a lower total restrictive practice event rate (incidence rate ratio [IRR] = 0.65, 95% CI [0.60, 0.69], *p* <.001) over a twelve-month intervention period. At a granular level, implementation sites had lower IRRs for seclusion and as-needed intramuscular psychotropics than controls; however, within-group rates fluctuated over the year. Two clusters of de-escalation responses and additional supplementary strategies (including stimulus reduction, music, and one-on-one staff time) were noted. The differential associations between clusters and the outcomes were insignificant.

**Conclusion:**

Despite mixed results, the evaluation offers support that structured therapeutic responding helps minimise restrictive practices, without evidence suggesting a substitution of one form of coercion for another.

## Introduction

De-escalation is the purposeful use of verbal, non-verbal, and relational strategies to interrupt emotional activation, address troubling behaviours, defuse potential interpersonal conflict, and eventually restore some sense of safety for all parties (1, 2). In public acute mental health units, it occurs in an inherently coercive context, where hospitalisation is increasingly involuntary (3, 4). Nurses are the first to respond to an escalation event among other professionals in a unit (5), although they are often working with limited specialist mental health training (6, 7), are instrumental in ensuring the continuity of service delivery (8), and are deploying primarily pharmacological treatments (9). In addition, nurses enforce legally sanctioned measures that restrict individuals’ civil liberties or contradict their expressed preferences (10). Seclusion, physical restraint, and forced medication typify these rights-limiting measures (11), widely recognised as carrying moral and relational costs for nurses (12). Globally, in response to these challenges, governments and broader care communities have called for reducing restrictive practices by promoting the development of relational capabilities and less coercive responses to escalation (13–16). However, training remains variable and under-resourced, with further evidence needed to strengthen sustained integration (17). Some training also continue to focus on ‘breakaway’ and restraint techniques (18), with comparatively less emphasis on relational approaches to de-escalation.

There are gaps in scientific knowledge bases that inform the development and implementation of de-escalation and other non-pharmacological interventions to reduce restrictive practices. Evidence syntheses are accessible on, for instance, staff training (19), restrictive practice review (20), and multi-component interventions, such as the Safewards (21). The intended impact of these interventions on restrictive practices has generally been favourable, although the strength of their associations is often limited or unassessed. Specifically, for Safewards, reporting of implementation fidelity has been inconsistent, with low or highly variable uptake of core components across intervention sites (22). On the other hand, sensory room and equipment (20) and risk assessment (21) have been linked to a mixed impact on reducing restrictive practices. Trauma-informed solutions, while promoted for recognising trauma and preventing re-traumatisation, have been critiqued by people with experience of using mental health services as often indistinguishable in practice, with symbolic commitments undermined by enduring power imbalances in coercive systems (23). Many of these interventions were developed and evaluated without considering: a) a broader range of disaggregated nurse-sensitive outcomes beyond Safewards’ ‘conflict’ and ‘containment’ measures (24), b) research bias-reducing measures, such as statistical controls for confounding (25), c) extended follow-up durations approaching one year (26), d) theories, models, and frameworks in implementation and evaluation (27), and e) the wisdom, understandings, and preferences of disempowered groups of people (e.g., Indigenous people) and people with experience of using mental health services (28) and of coercion (29). Moreover, no attempt has been made to identify which specific de-escalation components, such as active listening and limit-setting, contributed most to the intervention’s overall impact on restrictive practice use (30). Initiatives are also needed to improve the content, scope, adaptation, implementation, evaluation quality, and impact of de-escalation training interventions (31).

### Early implementation and support for the Safe Steps

In an adult mental health inpatient unit in regional New South Wales (NSW), Australia, a four-step approach to de-escalation, with tiered levels of complexity, was developed by a clinician in response to persistently high rates of physical restraint and seclusion, as well as the lack of clear, consistent guidance available to nurses on de-escalation (32). This approach was intended to bring order to how nurses hold space for the person in distress to explore their situation, feel listened to, discuss next steps collaboratively, and make sense of what actions may support exercising the following steps when the conversation is over. An earlier cluster analysis and between-cluster comparison, using a separate dataset, for discerning the most active co-occurring relational capabilities employed by nurses in the acute inpatient unit provides support for the structure and progression of the four-step approach (33). The steps were anchored in values of emotional intelligence, trauma-informed care, and personal recovery, which were emphasised in training on the approach. A separate feature analysis offers evidence for the importance of additional values, including cultivating situational and contextual awareness in early signs work and promoting autonomy-preserving de-escalation in inherently restrictive environments (34). These values were made explicit in tailoring practice through feedback loops in restrictive practice reviews. A separate weighted before-and-after analysis indicated that these restrictive practice reviews, conducted by a nurse unit manager with nurses as review participants and nurses’ practice of relational capabilities as a core review content, were associated with a significant reduction in seclusion in the acute inpatient unit (35). Taken together, the (i) four-step approach to therapeutic responding, implemented with the support of a (ii) phased training on the approach and the underlying values, and (iii) regular restrictive practice review meetings, comprise the Safe Steps for De-escalation, or simply the *Safe Steps*.

Given the observed reduction in seclusion, the initial proponents and evaluators of the Safe Steps considered this finding as preliminary evidence and expanded the evaluation to include a broader set of restrictive practice outcomes. The Safe Steps components were subsequently refined for implementation in a full-scale intervention evaluation. The hypothesised causal pathway from the Safe Steps implementation to reduced restrictive practice use, estimated using inverse probability weighting under standard causal inference assumptions, was considered to happen through changes in nurses’ relational capabilities, targeted to increase emphasis on developing and maintaining therapeutic relationships and self-management of people receiving care.

## Materials and methods

### Study design and participants

This paper reports findings from a larger study, employing a mixed concurrent control design, aimed at evaluating and implementing the Safe Steps. This paper addresses the study’s first objective, which sought to establish the intervention’s impact on total restrictive practice events (defined as the sum of events of seclusion, physical restraint, and as-needed intramuscular [IM] psychotropics), total restrictive practice durations (i.e., the sum of seclusion and physical restraint durations), and physical injury events (defined as the sum of bodily injury events incurred by unit staff, visitors, and people receiving care; this outcome does not include those that result from self-harm). The hypothesis tested was that the implementation units would show lower and more significant reductions in incidence rate ratios (IRRs) of total restrictive practice events, in model-derived estimates of total restrictive practice durations, and in IRRs of physical injuries, compared to control groups and within-group baseline. As an ancillary undertaking, the change mechanisms of the Safe Steps were explored through cluster analysis and by reviewing responses from the larger study’s qualitative assessment of process (experience and perspective of nurses and people receiving care through focus groups and interviews, respectively; to be published later).

The evaluation was informed by a researcher-designed implementation and evaluation framework, based on pragmatism (36) and complexity intervention research guidance (37). This framework posits that change is unique for every mental health service, grows through feedback, emerges unexpectedly, depends on shared relationships between agents of change, and needs to respect people’s choices. An overview of evidence syntheses has been a scaffold to this frame, indicating that the success of solutions in the field is hugely influenced by the contexts in which they were implemented (38). As used in health services research, complexity theory is a perspective that gives primacy to the relationships between agents of change in a service as influential in the successful delivery and evaluation of any service change (39). To support the assessment of selective reporting bias, the intervention, the proposed change mechanisms, the researcher-made implementation and evaluation framework, and the protocol for the larger study are described elsewhere (40).

Implementation was at the unit level in three sites in two NSW local health districts (LHD), with three control sites. An LHD is a regional health authority responsible for delivering public health services within a defined geographic area (41). Outcome comparisons were made between and within these six declared acute adult mental health inpatient units in public hospitals. Clinical input, each site’s readiness, and local governance processes determined the selection of implementation and control sites. The implementation sites had a combined bed capacity of 75, which included ten for high observation. The control sites had a total of 84 beds but had no high observation areas; one site lacked a seclusion room entirely. Each unit operates 24 hours a day, 7 days a week, and varies in care models, restrictive practice rates, peer worker integration, and restrictive practice review processes. These units have the legal authority to admit and treat people involuntarily, and are situated in NSW where 46% of public hospital acute mental health service hospitalisations for 2022 to 2023 were involuntary (4). Given the pragmatic, complexity-informed design of the current evaluation, which was intended to balance practicality and robustness in methodologies, inverse probability weighting was applied. This advanced statistical technique supports re-balancing the observed groups on measured characteristics to approximate the balance that randomisation would otherwise achieve (42).

At the time of the implementation, no other restrictive practice-reduction initiatives were being evaluated or trialled within the participating sites. Site implementation leads were proactive in informing the research team of any initiatives that might otherwise have conflated with the current evaluation. There were proposed initiatives identified towards the end of the Safe Steps evaluation, but implementation was postponed to avoid overlap. Furthermore, there was no standardised or structured approach regarded as a ‘go-to’ model for de-escalation across sites. In contrast, the Safe Steps was intended to bolster the uptake of relational approaches to de-escalation, strengthen reflective practice, and introduce proper documentation of relational capabilities. Foremost, it was designed to provide structured education on both theory and real-world examples of relational de-escalation.

The evaluation was undertaken across four time points per site over 12 months to support the emergence of short-, medium-, and long-term changes associated with the phased training on the Safe Steps. This observation period was informed by *a priori* Monte Carlo simulations, using the simr package version 1.0.8 (43) and the dataset from the initial implementation of the restrictive practice review meetings. It was estimated that, with *a* = 0.05 and a 20% attrition buffer, 3.60 to 12 months of data collection would be required to detect outcome-specific reductions in seclusion, physical restraint, and Code Black—a hospital emergency code called to summon the presence and support of security personnel in response to personal safety threats (44), with 85% to 99% power based on Poisson mixed-effects models. Effect size assumptions for the simulations aligned with findings from the NSW Safewards evaluation (45), which was judged as high in methodological quality (21) and shares a similar geographical context.

Ethical approvals for the implementation and evaluation were obtained from Human Research Ethics Committees in an LHD (2023/PID00297 - 2023/ETH00272) and a university (2023/069) in NSW, Australia. The implementation and evaluation were undertaken with oversight from a project steering committee.

### Procedures

An opt-in approach was followed in the one-year, multi-part implementation, with each participating site deciding its own start date based on readiness and the value of autonomy and self-organisation. Implementation began on the 1^st^ of March 2024 at the first site and concluded on the 15^th^ of April 2025, following the completion of the one-year follow-up at the third site. The baseline period covered the year preceding each site’s implementation start date. After site-specific administrative preparations and train-the-trainer sessions, implementing the Safe Steps into routine practice commenced with in-person training on the four-step approach and complementary online multimedia modules delivered to all participating nursing staff. The online modules, with theoretical texts and diagrams, podcasts-like audios, and short video clips, were co-developed with peer workers, Aboriginal Elders and health leads, and interdisciplinary stakeholders (see **Table 1** for the key contents).

**Table 1:** Contents of the complementary online multimedia module on the Safe Steps.

Module	Lessons (No. & Duration)	Key content
1	3 lessons (~30 min each)	Introduction to the Safe Steps framework; impact of power imbalances; reflection on cultural beliefs and behaviours; understanding trauma and emotional dysregulation.
2	4 lessons (15–30 min each)	Collaborative de-escalation approaches; role of lived experience and peer workers; principles of culturally safe and respectful care for Aboriginal and diverse communities.
3	3 lessons (20–30 min each)	Emotional intelligence skills (e.g., empathy, affect labelling, refocusing); trauma-informed care with concepts like neuroception.
4	2 lessons (~30 min each)	The four-step Safe Steps framework; using structure, shared language, and safety checkpoints to guide flexible, coordinated real-time responses.

A suite of implementation strategies was employed to embed the values emphasised in the Safe Steps into daily working practice, promote the uptake of the steps, and minimise the bias arising from deviation from the intended intervention. Implementation was supported by nurse educators and reinforced through applications of paper posters, pull-up banners, email signature banners, pocket cards on the four-step approach, coffee vouchers, and weekly routine site visits and meetings. All nursing staff were trained to promptly record contemporaneous event-level data through a de-escalation log to minimise recall bias. De-escalation log collection began on the first day of the implementation. Implementation fidelity was tracked once per time point per site, using an adapted observational checklist.

Restrictive practice review meetings were offered to, and received by, nurse participants from the third month of the implementation year, to allow for sufficient log data collection. The reviews were undertaken monthly during the units’ one-hour in-service sessions. Nurse educators used presentation slides on aggregated de-escalation log data to support ongoing reflection, feedback, and learning, focusing on celebrating the nurse participants’ practice of relational capabilities. Word clouds on de-escalation triggers and plots on trends and patterns in log completion, restrictive practices, the Safe Steps, other de-escalation techniques, and co-de-escalation across levels of situational aggression were employed.

### Outcomes

Outcomes measured were the rates of total restrictive practice events, the estimates of total restrictive practice durations, and the rates of physical injury events. These were prospectively gathered from routinely maintained administrative datasets, including the incident management system (ims+) for physical injury and Code Black events, electronic medication records (eMeds) for the IM psychotropic administrations, and local unit registers on seclusion and physical restraint events and durations. All seclusion and physical restraint events and durations undergo data checks and cross-referencing with ims+, as part of legally mandated reporting to the Australian National Seclusion and Restraint Database (46). Similarly, all incidents recorded through ims+ are reviewed and flagged for action by service management (47). At the same time, eMeds offers digital oversight associated with fewer medication errors, particularly in rural NSW (48). Routinely collected administrative data for the outcomes were intended to minimise performance and detection bias risks.

Seclusion and physical restraint events and durations were chosen as evaluation outcomes, as they are nationally mandated performance indicators in Australian public acute mental health services (46). Seclusion refers to the act of placing a person alone in a room from which they are unable to leave independently, whereas physical restraint involves staff using their hands or body to restrict a person’s movement (46). On the other hand, Code Black was excluded *a priori* from hypothesis testing, yet was analysed and reported to support information development (49). As-needed IM psychotropics, while not a formal service performance indicator, were included due to their restrictive nature, particularly given their parenteral route, rapid onset, sedative formulation, and the influence nurses may have on their administration in practice. Sometimes, as-needed IMs are given involuntarily, often in conjunction with physical restraint. Included drug classes in this evaluation were typical and atypical antipsychotics (i.e., droperidol, haloperidol, olanzapine, ziprasidone, zuclopenthixol acetate) and benzodiazepines (i.e., lorazepam and midazolam). The selection of these psychotropics as evaluation outcomes was informed by a review of the academic literature (50–52), clinical inputs, and the participating sites’ guidelines for the care and preliminary sedation requirements of people who present with acute behavioural disturbance. Administration of zuclopenthixol acetate requires psychiatrist approval and was included, given its sedative profile and potential to restrict autonomy (53). Furthermore, nurses also play a role in suggesting or withholding zuclopenthixol acetate use and monitoring its effects after administration (54).

Nurse-recorded de-escalation logs were collected to document incident details (including triggers of the de-escalation), de-escalation contexts (including a clinician-made 6-point situational aggression scale), and de-escalation practices (e.g., the Safe Steps, redirection, sensory modulation; see page 5 in **Supplementary Materials** for descriptions). These logs were the primary data source for exploring key co-occurring responses contributing to the Safe Steps’ impact, and for describing the administratively recorded sex and mental distress diagnoses of individuals receiving care, who were involved in the de-escalation events. Implementation fidelity was also tracked using an adapted checklist from the Safewards (55). Items 1 to 5 were about the core elements of the Safe Steps in practice, including i) regular reflective practice, ii) nurses’ use of emotional intelligence capabilities, iii) identification and response to early warning signs of emotional distress, troubling behaviours, interpersonal conflict, and potential restrictive practice use, iv) engagement with the structured de-escalation framework, and v) application of de-escalation techniques.

### Data analysis

Outcome events and durations were aggregated at the day level to serve as the unit of analysis. Aggregation was intended to support the determination of missing data (55) and imputation for outcomes without a true reference value. No exclusion criteria were applied to the outcome events and durations, provided they occurred within the defined study period. Administrative and log data were anonymised before analysis. The primary analysis excluded the log-derived demographic information of people and nurses involved in de-escalation. To minimise measurement bias, the first author remained blinded to the identities of the people and nurses involved in restrictive practice use and de-escalation events. The authors also held no employment affiliation with any participating sites.

A range of *a priori* analyses was conducted across three comparison types: i) between-group differences across intervention and control sites before and during the Safe Steps implementation, ii) within-group changes at the implementation sites, and iii) exploratory outcome associations with de-escalation response clusters, determined through a cluster analysis. All quantitative analyses were undertaken using R version 4.2.3 (56) and RStudio version 2023.12.1 + 402 ‘Ocean Storm’ (57), while visuals were plotted through matplotlib version 3.9.3 (58, 59). within JupyterLite version 0.6.3 (60). Hypotheses were tested using incidence rate ratios (IRRs) and model-derived estimates (*β*) at *p* <.05. For descriptive reporting, frequencies of events and durations and rates per 1,000 occupied bed days were used. With the guidance on handling missing data for randomised clinical trials (61), missing log data were addressed via multiple imputation through the mice package (62) under both missing at random and missing not at random assumptions.

*A priori* generalised linear mixed models (GLMMs) were used for between- and within-group comparisons for count-based outcomes (i.e., restrictive practice events, physical injury and Code Black). In contrast, *a priori* linear models (LMs) were used for continuous outcomes (i.e., restrictive practice duration). Model family selection for GLMMs (i.e., Poisson, negative binomial, or zero-inflated) was informed primarily by outcome distribution characteristics, including tests for over-dispersion and the presence of excess zeros. In instances where residual normality or independence assumptions were violated in the LMs, linear mixed-effects models (LMMs) were applied. A range of *a priori* random effects was considered in model specification, which included the implementation unit, calendar day, month, year, and study day index. All models underwent diagnostics, including convergence checks and overall model fit indices. The coefficients of determination (R²) were calculated using the delta method (63). Parsimony and explanatory strength were considered in the final selection among competing models (see page 2 in the **Supplementary Materials** for the final model specifications).

*A priori* inverse probability weighting (IPW) was applied using the ipw package (64) to address potential baseline imbalance and confounding. *A priori* covariates in the IPW models included the average harm scores of daily event aggregates, the number of high observation beds, the presence of seclusion rooms, and confounds, i.e., physical restraint and presence of security personnel, summoned during Code Black activations, are interrelated (65). A harm score was used to quantify the average impact severity of the daily aggregate of events (47); its inclusion was informed by a feature analysis that indicated event immediacy as an algorithmically, statistically, and epistemologically important restrictive practice driver (34). The unit without the seclusion room was removed from the weighted comparisons for the seclusion outcome. Relevant guidelines were considered for employing and reporting IPW (42).

To identify the most active co-occurring patterns of nursing de-escalation practices associated with the Safe Steps’ overall impact, *a priori* hierarchical cluster analysis was conducted on de-escalation log data using Canberra distance, average linkage, silhouette plots, and the cophenetic correlation coefficient (CCC) for determining the optimal clustering solution. These methods are a collection of tools for determining how best to group daily practice event patterns into clusters. Each daily aggregate of events during the implementation year was assigned to one of the emerging clusters. Median values and interquartile ranges were then calculated to describe the emerging clusters. The clusters were then included as fixed effects in GLMM, LM, or LMM models to assess their differential associations with restrictive practice events and duration, physical injuries, and Code Black. Model diagnostics and assumption verifications have been applied to the mixed models. Consistent with the aforementioned guidance for missing data treatment and this evaluation’s published protocol, no sensitivity analyses in relation to multiple imputation of missing log data were undertaken to avoid disrupting the clustering structure underpinning the fixed effect for the mixed modelling subsequent to the cluster analysis.

*A priori* fidelity assessment was undertaken by counting the conduct of core Safe Steps components across implementation sites. As no established norms exist to define full or partial implementation, higher counts on items 1 to 5 of the adapted checklist were interpreted as indicative of more robust implementation. In comparison, lower counts suggested partial or minimal uptake. Fidelity scores were summarised descriptively.

## Results

There was no attrition among participating acute mental health units. Following the services’ check and balance systems, no outcomes from administrative data were considered missing. After a year of implementation, most days had at least one de-escalation log (n_days_/N_days_ = 324/365; 89%). This response rate equates to 2,955 logs across three implementation sites. When disaggregated into four time points, the response rate was 80% (n_days_ = 73) at the first time point, 96% (n_days_ = 87) at the second, 92% (n_days_ = 84) at the third, and 87% (n_days_ = 80) at the fourth. Total missing log data (n_days_ = 41; 11%), excluding demographics, was imputed.

Of the recorded de-escalation events, 81% showed a decrease in situational aggression on a 6-point scale, with an average reduction of 1.20 points. The modal change was a 1-point reduction. Most events on the scale were reported as verbal aggression (level 3; 48%) and agitation (level 2; 30%), with 74% of events directed towards nurses. Discounting repeated responses involving the same individual, de-escalation mainly involved male individuals (58%). In comparison, schizophrenia (33%), schizoaffective disorder (13%), and drug-induced psychosis (13%) were the most common primary diagnoses recorded (see **Table 2**).

**Table 2:** Key characteristics of inpatients involved in de-escalation events.

Item	n (N = 424)	%
Sex		
Female	158	37%
Male	246	58%
Unclassified	20	5%
Primary Diagnosis or Presentation		
Schizophrenia	141	33%
Schizoaffective Disorder	57	13%
Drug-induced Psychosis	53	13%
Bipolar Affective Disorder	47	11%
Borderline Personality Disorder	25	6%
Adjustment Disorder, Alcohol Dependence, Antisocial Personality Disorder, Anxiety Disorder, Autism Spectrum Disorder, Cluster B Personality Disorder; Treatment Resistant Depression, Complex Post-traumatic Stress Disorder, Delirium, Delusional Disorder, Dementia, Eating Disorder, Intellectual Disability, Major Depressive Disorder, Post-traumatic Stress Disorder, Psychotic Depression, Substance Use Disorder, Traumatic Brain Injury	41	10%
Psychosis	20	5%
Acute Stress Reaction, Agitated Behaviour, Cannabis Withdrawal, Family Conflict, First Episode Psychosis, Hypomania, Mania, Manic Psychosis, Methamphetamine Intoxication, Organic Brain Syndrome, Paranoid Psychosis, Personality Vulnerabilities, Recurrent Psychotic Episodes, Substance Withdrawal, Suicidal Ideation	23	5%
Unknown	17	4%

To preserve anonymity, frequencies of demographic information with fewer than 10 samples were aggregated with adjacent strata in the summary statistics. The frequencies shown above discount repeated responses involving the same individuals.

A good covariate balance was achieved on harm scores and the known confounds (i.e., physical restraint to Code Black, and vice versa). However, consistent imbalances were noted for the infrastructure-related covariates (i.e., the presence of a dedicated seclusion room and access to high-observation beds), which showed residual differences after weighting, specifically for between-group comparisons before and during the Safe Steps implementation. Sample sizes after adjustment remained sufficient to support outcome modelling. Love plots for the completed IPW are available on page 3 of the **Supplementary Materials**.

### Between-group comparisons

**Table 3** shows the IRRs for the intervention effect on event rates and the *β* for the mean difference in event duration (in minutes) between implementation and control sites at one-year baseline and one-year implementation period. Compared to the control sites, the intervention sites showed during implementation significantly lower rates of seclusion (IRR = 0.77, 95% CI [0.59, 0.99], *p* = .04), as-needed IM psychotropics (IRR = 0.58, 95% CI [0.54, 0.63], *p* <.001), and total restrictive practice events (IRR = 0.65, 95% CI [0.60, 0.69], *p* <.001). There were no significant differences with the other outcome measures; however, most stayed directionally favourable for the implementation sites. Consequently, as the restrictive practice event rate at the implementation sites was significantly lower than the control sites, there is equivocal support for the between-group comparison subset of the hypothesis.

**Table 3:** Between-group comparison outcomes at one-year baseline and one-year implementation.

Outcome	Control	Implementation	IRR/β Control vs Implementation	95% CI	*p*	ICC	Marginal R^2^	Conditional R^2^
	n	n						
Baseline								
Total Restrictive Practice Events	2,420	1,395	0.54	0.19 – 1.57	0.26	0.34	0.10	0.57
*Seclusion*	109	96	0.50	0.40 – 0.63	<0.001	0.35	0.01	0.13
*Physical Restraint*	165	133	0.56	0.46 – 0.69	<0.001	0.64	0.01	0.16
*IM Psychotropic use*	2,146	1,166	0.50	0.16 – 1.60	0.24	0.39	0.11	0.58
Physical Injury	12	11	0.56	0.27 – 1.17	0.13	0.94	0.45	0.67
Code Black	200	183	0.45	0.34 – 0.60	<0.001	0.81	0.05	0.63
Total Restrictive Practice Duration*	793	751	-15.87	-63.18 – 31.43	0.51	0.01	5.66 × 10^-4^	0.01
Seclusion Duration*	787	744	-32.86	-60.57 – -5.15	0.02	0.01	1.89 × 10^-3^	0.01
Physical Restraint Duration**	314	457	- 0.05	-0.48 – 0.38	0.83	3.44 × 10^-3^	4.19 × 10^-5^	3.49 × 10^-3^
During Implementation								
Total Restrictive Practice Events	1,940	1,028	0.65	0.60 – 0.69	<0.001	0.23	0.07	0.44
*Seclusion*	69	59	0.77	0.59 – 0.99	0.04	0.50	1.30 × 10^-3^	0.19
*Physical Restraint*	128	121	0.83	0.68 – 1.03	0.09	0.69	9.96 × 10^-4^	0.15
*IM Psychotropic use*	1,743	848	0.58	0.54 – 0.63	<0.001	0.21	0.10	0.40
Physical Injury	7	10	0.99	0.42 – 2.34	0.98	0.91	3.93 × 10^-6^	0.07
Code Black	138	121	1.03	0.86 – 1.23	0.77	0.49	2.22 × 10^-5^	0.23
Total Restrictive Practice Duration*	487	394	- 8.02	-39.39 – 23.35	0.62	0.02	4.34 × 10^-4^	0.03
Seclusion Duration*	484	388	-17.10	-37.96 – 3.76	0.11	0.02	1.60 × 10^-3^	0.02
Physical Restraint Duration**	186	354	0.13	-0.02 – 0.28	0.08	0.09	1.66 × 10^-3^	0.09

β, estimate for continuous data (i.e., duration); *, n is hours, but β is in minutes; **, n and β are in minutes; CI, confidence interval; IM, intramuscular; IRR, incidence rate ratio for count data (i.e., non-duration outcome measures); ICC, intraclass correlation coefficient; R^2^, coefficient of determination. The n reflects the number of observations included in each model. The estimates (IRRs and beta coefficients) are derived from weighted models; however, the n values themselves are unweighted.

If the comparison was to be extended to the drug classes of as-needed IM psychotropics at a frequency level, the IM antipsychotics (discounting zuclopenthixol acetate) in the implementation sites (n = 307) were 61.2% lower than in the control sites (n = 791). In contrast, the use of IM benzodiazepines in the implementation sites (n = 366) was 59.1% lower than in the control sites (n = 895). When zuclopenthixol acetate was added to the IM antipsychotics, the implementation site recorded 482 administrations, which is 43.2% lower than in the control sites (n = 848).

### Within-group comparisons

Out of the 36 within-group comparisons (see the number of IRRs and *β* in **Table 4**) conducted, six comparisons reached statistical significance. Compared to the one-year baseline, the implementation sites showed significant reductions in seclusion after time points 1 (IRR = 0.43, 95% CI [0.21, 0.88], *p* = .02) and 3 (IRR = 0.44, 95% CI [0.22, 0.89], *p* = .02). Significant reductions were also noted in as-needed IM psychotropics at time points 1 (IRR = 0.73, 95% CI [0.61, 0.87], *p* <.001) and 3 (IRR = 0.77, 95% CI [0.64, 0.92], *p* = .004), and in total restrictive practice events at time points 1 (IRR = 0.75, 95% CI [0.64, 0.88], *p* <.001) and 3 (IRR = 0.80, 95% CI [0.69, 0.94], *p* = .005). For the outcome measures with no significant changes, the directions of the effects were mostly downward. As no significant reductions in outcomes were observed at the fourth time point versus at baseline, the within-group comparison component of the hypothesis is not considered as supported.

**Table 4:** Within-group comparison: one-year baseline and quarterly time points.

Outcome	n	IRR/*β* Baseline vs Time Point	95% CI	*p*	ICC	Marginal R^2^	Conditional R^2^
Total Restrictive Practice Events							
Baseline	1,395	1.00 (ref)			0.36	0.01	0.46
Time point 1	227	0.75	0.64 – 0.88	<0.001			
Time point 2	278	0.89	0.77 – 1.02	0.10			
Time point 3	236	0.80	0.69 – 0.94	0.005			
Time point 4	287	0.97	0.85 – 1.12	0.71			
Seclusion							
Baseline	96	1.00 (ref)			0.31	0.01	0.09
Time point 1	10	0.43	0.21 – 0.88	0.02			
Time point 2	19	0.83	0.47 – 1.47	0.53			
Time point 3	10	0.44	0.22 – 0.89	0.02			
Time point 4	20	0.87	0.50 – 1.53	0.64			
Physical Restraint							
Baseline	133	1.00 (ref)			0.32	1.85×10^−3^	0.11
Time point 1	27	0.84	0.51 – 1.39	0.50			
Time point 2	25	0.82	0.50 – 1.36	0.45			
Time point 3	38	1.29	0.82 – 2.01	0.27			
Time point 4	31	1.09	0.69 – 1.74	0.71			
IM Psychotropic use							
Baseline	1,166	1.00 (ref)			0.43	0.01	0.47
Time point 1	190	0.73	0.61 – 0.87	<0.001			
Time point 2	234	0.88	0.76 – 1.04	0.13			
Time point 3	188	0.77	0.64 – 0.92	0.004			
Time point 4	236	0.90	0.77 – 1.05	0.20			
Physical Injury							
Baseline	11	1.00 (ref)			0.11	0.05	0.11
Time point 1	1	0.38	0.05 – 3.18	0.37			
Time point 2	1	0.38	0.05 – 3.12	0.37			
Time point 3	3	1.29	0.33 – 5.04	0.72			
Time point 4	5	2.29	0.70 – 7.47	0.17			
Code Black							
Baseline	183	1.00 (ref)			0.77	0.01	0.35
Time point 1	36	0.77	0.51 – 1.15	0.19			
Time point 2	32	0.72	0.47 – 1.09	0.12			
Time point 3	25	0.63	0.39 – 1.01	0.054			
Time point 4	28	0.66	0.43 – 1.03	0.07			
Total Restrictive Practice Duration*							
Baseline	752	0 (ref)			0.01	1.97×10^−3^	0.01
Time point 1	57	-27.82	-59.78 – 4.13	0.09			
Time point 2	157	- 5.08	-37.18 – 27.02	0.76			
Time point 3	76	-22.73	-54.84 – 9.38	0.17			
Time point 4	105	-15.33	-47.44 – 16.77	0.35			
Seclusion Duration*							
Baseline	744	0 (ref)			0.01	1.96×10^−3^	0.01
Time point 1	56	-27.63	-59.54 – 4.28	0.09			
Time point 2	155	- 5.01	-37.06 – 27.04	0.76			
Time point 3	74	-22.82	-54.88 – 9.25	0.16			
Time point 4	104	-15.23	-47.28 – 16.83	0.35			
Physical Restraint Duration**							
Baseline	457	0 (ref)			0.01	7.65×10^−4^	0.01
Time point 1	59	- 0.15	-0.60 – 0.30	0.52			
Time point 2	90	- 0.01	-0.47 – 0.44	0.95			
Time point 3	129	0.21	-0.24 – 0.67	0.36			
Time point 4	76	- 0.05	-0.51 – 0.40	0.81			

β, estimate for continuous data (i.e., duration); *, n is hours, but β is in minutes; **, n and β are in minutes; CI, confidence interval; IM, intramuscular; IRR, incidence rate ratio for count data (i.e., non-duration outcome measures); ICC, intraclass correlation coefficient; R^2^, coefficient of determination. The n reflects the number of observations included in each model. The estimates (IRRs and beta coefficients) are derived from weighted models; however, the n values themselves are unweighted. Decimal values for n of duration outcomes were rounded to whole numbers, which may result in minor discrepancies when summing non-composite duration outcomes. The IRR of the outcome measures for baseline has been considered as 1, while the β for the baseline is 0. Values represent baseline and four subsequent time points, with the corresponding number of restrictive practice events (e.g., 1,395 at baseline for total restrictive practice events is followed by 227, 278, 236, and 287 at the four time points). R² values are from the overall mixed-effects models for a given outcome and are not estimated separately for each time point comparison for a particular outcome.

Including zuclopenthixol acetate under IM antipsychotics, the total number of antipsychotic administrations across time points 1 to 4 was 482, representing a 28.5% reduction compared to the one-year baseline (n = 674). For IM benzodiazepines, administrations across time points 1 to 4 totalled 366, which is a 25.6% reduction from baseline (n = 492). Excluding zuclopenthixol acetate from the IM antipsychotic category, the implementation sites recorded 307 antipsychotic administrations across time points 1 to 4, being a 39.3% reduction compared to baseline (n = 506).

### Cluster analysis and between-cluster comparisons

**Figure 1:**
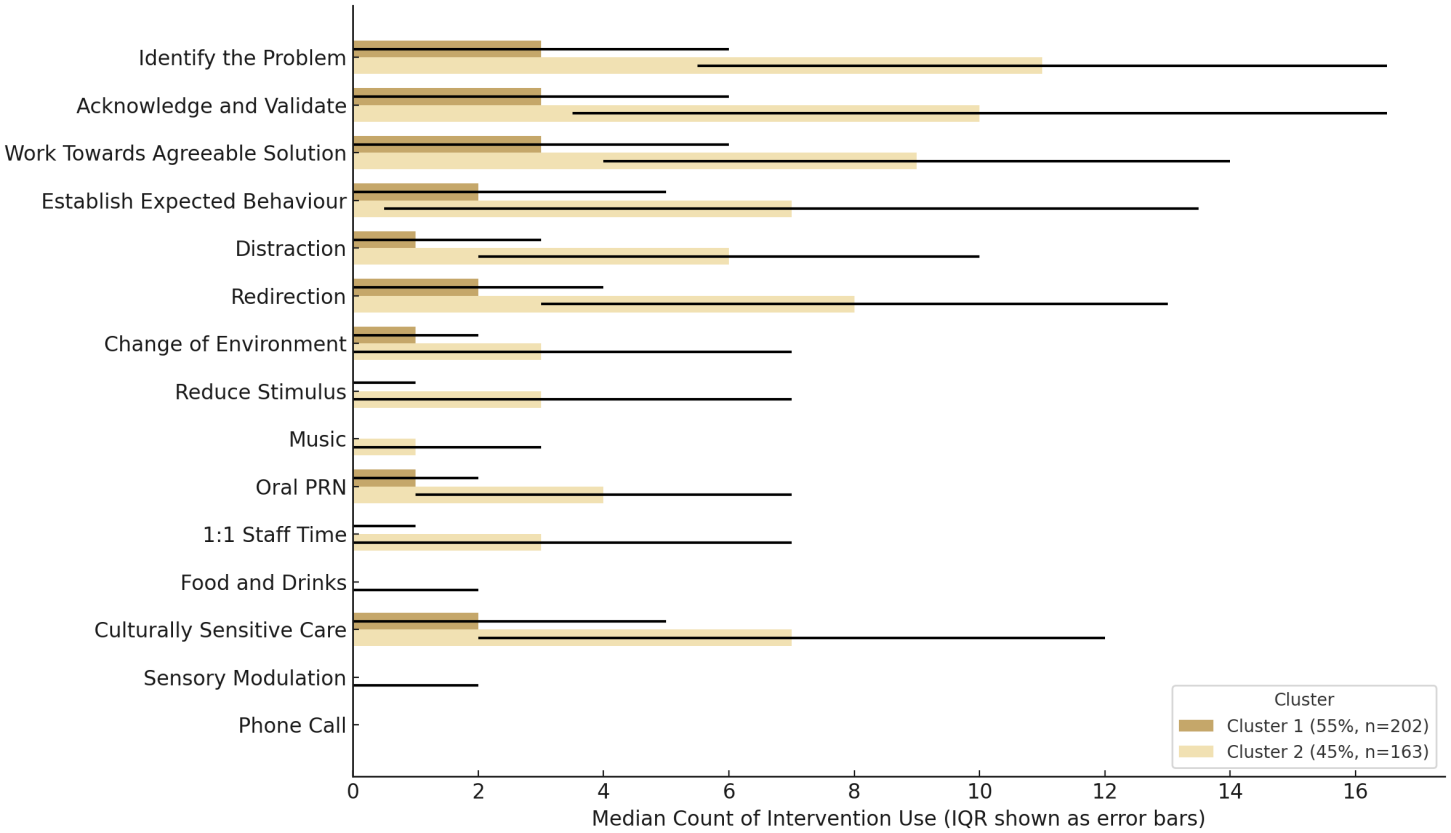
Median and interquartile ranges (IQR) of de-escalation practices across groups of co-occurring responses.

The silhouette plot showed that two clusters of co-occurring de-escalation practices provided the optimal solution (see page 4 of the **Supplementary Materials**), which is supported by a CCC of 0.79 that reflects a stable clustering. Consequently, each daily aggregate of log data was assigned a cluster. In 202 days (55%) of the implementation year, nurses responded to prompts of de-escalation through the Safe Steps and a range of co-occurring practices (Cluster 1), including distraction, redirection, change of environment, as-needed oral medication response to behavioural disturbances, and culturally sensitive care (see **Figure 1**). For the rest of the year, in addition to the co-occurring practices in Cluster 1, nurses also applied one-on-one staff time, reduced stimulus, and music, while using the Safe Steps variably (Cluster 2). Cluster 2 is higher by three total restrictive practice events per 1,000 occupied bed days than Cluster 1, and by 11 hours of total restrictive practice duration per 1,000 occupied bed days (see **Table 5**). However, the difference between Clusters 2 and 1 on all outcome measures is not statistically significant. At a frequency level, the Safe Steps was used most often at time point 2 of the implementation year, followed by time points 3, 4, and 1.

**Table 5:** Between-cluster comparison outcomes.

Outcome	Cluster 1	Cluster 2	IRR/*β* Cluster 1 vs 2	95% CI	*p*	ICC	Marginal R^2^	Conditional R^2^
	RPO	RPO						
Total Restrictive Practice Events	43	46	1.04	0.84 – 1.28	0.71	0.07	4.18 × 10^-4^	0.62
*Seclusion*	2	3	1.36	0.79 – 2.34	0.27	0.18	0.01	0.03
*Physical Restraint*	5	6	1.18	0.78 – 1.80	0.44	0.03	4.28 × 10^-3^	0.01
*IM Psychotropic use*	36	37	1.02	0.84 – 1.25	0.84	0.09	1.26 × 10^-4^	0.50
Physical Injury	1	0	0.37	0.07 – 2.12	0.27	0.95	0.05	0.43
Code Black	5	6	-	-	-	-	-	-
Total Restrictive Practice Duration*	12	23	41.23	-13.38 – 95.84	0.14		0.01	
Seclusion Duration*	12	22	40.90	-13.49 – 95.29	0.14		0.01	
Physical Restraint Duration**	13	18	0.33	-0.36 – 1.02	0.35		0.002	

β, estimate for continuous data (i.e., duration); *, n is hours, but β is in minutes; **, n and β are in minutes; CI, confidence interval; IM, intramuscular; IRR, incidence rate ratio for count data (i.e., non-duration outcome measures); ICC, intraclass correlation coefficient; R2, coefficient of determination; RPO, rate per 1, 000 occupied bed days. Code black model did not converge.

### Fidelity checks

Assessment of fidelity to intended intervention was limited, as no checklists were completed during the first and second implementation time points, due to pending ethical approval for the modified checklist, which was obtained towards the end of the second time point. Checklists were then gathered for all three sites at the third and fourth time points, with multiple submissions at some sites (n = 4 for time point three; n = 8 for time point four). All gathered checklists indicated full implementation of the Safe Steps, with all items from 1 to 5 marked as completed.

## Discussion

A year-long pragmatic implementation was undertaken across three acute mental health units in NSW, Australia, to assess the impact of the Safe Steps, a structured approach to therapeutic responding aimed at reducing the use of restrictive practices during events of emotional distress, troubling behaviours, and interpersonal conflict. This complex intervention research reflects multidisciplinary collaboration, sustained engagement from nurse participants, and the support of all participating inpatient units, with none withdrawing from the study. This collective effort helped determine the association between the Safe Steps implementation and lower total restrictive practice events, seclusion, and as-needed IM psychotropic use, compared to control sites, as well as other outcomes in which the implementation had no impact.

This evaluation fills several methodological gaps. A recent review on de-escalation training interventions found that much evaluative research was non-randomised, uncontrolled, and at serious risk of selective reporting bias (31). Many lacked preregistered protocols, statistical controls for confounding, blinded outcome analyses, *a priori* model-based power analyses, and detailed outcome reporting. Only one study examined forced medication, but no significant reduction was observed (66). Recent clustered randomised controlled trials (CRTs) have also narrowly focused on aggression, aggression severity, and physical restraint outcomes (67, 68). Similar concerns are evident in restrictive practice-reduction programmes beyond de-escalation (22, 25), with little attention given to discerning the most active intervention components (21, 69) and anchoring evaluations and implementations in frameworks (27, 70). In contrast, the current evaluation addressed these limitations.

During implementation, intervention sites had lower as-needed IM psychotropic use than controls, with no baseline between-group difference, suggesting the noted change may reflect an intervention-related effect. This finding contributed to the significantly lower total restrictive practice events during implementation, compared to controls. This finding aligns with earlier Safewards studies that included forced medication in the measure of total containment events (45, 55); nonetheless, it is unclear in these earlier studies as to which specific outcome drove the overall impact, making practical interpretations difficult. Beyond the de-escalation focus, hospital-wide interventions (i.e., open door policy and architectural modernisation of facilities) have shown comparable forced medication reductions (71, 72). However, they were without controls and multiple time points that help separate the intervention effect from natural outcome fluctuations. In contrast, this evaluation had four time points that helped identify significant within-group reductions in seclusion, IM use, and total restrictive practice event rates at months three and nine. Interestingly, these improvements contrasted the lack of change in seclusion and forced medication in a CRT for an evidence-based training (73), suggesting the potential positive difference that comes with inpatient-nurse relationship-building approaches like the Safe Steps.

There is a likely untracked increase in other potentially restrictive practices, particularly in as-needed oral medication use. This emergence is possible, given that clinical guidelines routinely recommend as-needed oral medication as an intermediate step between verbal de-escalation and IM sedation (74, 75). However, the cluster analysis findings, expert opinions, and a focus group insight suggest a more nuanced interpretation. Firstly, response clusters showed that, although as-needed oral medication had been used throughout the year, their median values were lower than those of the Safe Steps, suggesting their use may reflect more of an embedded stepped-care practice, rather than over-reliance. Secondly, unlike IM sedation, which is intrusive by virtue of its administration route, as-needed oral medication often involves a degree of choice (76), especially when voluntarily accepted. However, it can be challenging to recognise whether an agitated person’s acceptance of as-needed oral medication is truly voluntary (77), especially when a refusal may lead to an IM administration. Thirdly, a focus group account in the larger study reported that nurses prioritised relational engagement over IM use in containing escalated events. These findings suggest that the Safe Steps implementation did not influence substituting one restrictive practice for another, but may have promoted autonomy-preserving responses. Nevertheless, future studies may consider tracking as-needed oral medication alongside as-needed IM use to strengthen evaluations.

What might explain the positive findings? As seen in the emerged response clusters, the Safe Steps was used throughout the implementation year. This evidence suggests that nurses’ demonstrated relational capabilities could have restructured the unit’s social environment (78). Implementing the Safe Steps could have legitimised proactive relational engagement that reduced response ambiguity in escalated events. As reported in nurse focus groups nested in the larger study, another mechanism could be that repeated de-escalation log use may have improved nurses’ skills in recognising early warning signs of pending coercion. A Norwegian pilot study that included aggression risk assessment training to reduce coercion suggested a similar mechanism (79). Thirdly, as also found through nurse focus groups in the larger study, the four-step approach and the log may have functioned as cues and prompts (80) that reminded nurses of their uptake of relational capabilities. From the first author’s perspective, the Safe Steps likely operated through two mechanisms: instructional guidance through training and performance feedback and encouragement through review meetings (35). While there may have been a multi-part architecture for change behind the Safe Steps implementation, the possibility of reverse causation cannot be excluded. Implementation sites with lower baseline rates of restrictive practices may have been more predisposed to implementing the structured approach to de-escalation, which could have reinforced less coercive responses to escalations. This possibility intersects with the difficulty of detecting floor effects in count data where there are no true reference values. Sites already practicing less coercive de-escalation and have relatively low event rates may have shown limited capacity for further measurable reductions.

Implementing the Safe Steps did not impact restrictive practice durations between and within groups. This finding is similar to that of an earlier randomised control trial in fifty-four German psychiatric units on coercion and violence prevention guidelines (81). Physical injury in this evaluation was also too infrequent for meaningful comparison, and local policies typically influence Code Black activations. External factors during implementation, such as the state-wide industrial actions by nurses and psychiatrists coinciding with the third and fourth research time points and the sporadic organisational restructuring in participating sites, may have dampened change. These factors are difficult to control, similar to the infrastructure-related imbalances seen after applying IPW. Nonetheless, it was a pragmatic decision to control only for specific confounds and covariates, as closing too many potential back doors for a more robust causal pathway (82) could have shifted the evaluation’s focus from the original intention of assessing intervention effectiveness. In addition, adhering to the planned statistical analyses helps prevent potentially spurious results that may arise from questionable measurement practices (83).

The infrastructure-related difference and the relationship between bed profile and restrictive practice use are not straightforward issues. In Victoria, Australia, elevated restrictive practice rates have been attributed to higher thresholds for acute mental health service access (84), rather than bed profile. By contrast, the introduction of high observation units in Ireland coincided with a reduction in restrictive practices (85). There is also support for the link between physical design features (i.e., more private space, greater level of comfort, and greater visibility on the unit) and reduced risk of seclusion in psychiatric and forensic units within the Netherlands (86). In terms of intervention evaluations, it is uncommon for evidence syntheses to detail matching methods used in primary evaluation studies in the field (38), with some researchers noting that their control groups were ‘matched’ to service type (87), rather than bed profile. This service type matching is still valuable, although high-observation areas share features with secure services, where restrictive practices are more systematically embedded (88). Indeed, selecting controls for intervention evaluation is complex (89). However, if the relationship to be privileged is that high-observation areas are linked to higher restrictive practice use (34, 90), then it is notable that nurses in the implementation sites with high observation beds may have responded less coercively to a larger number of, and arguably more challenging, escalations there, suggesting the robustness of this evaluation’s between-group comparison findings.

The cluster analysis showed that Cluster 1, with no one-on-one staff time but consistent Safe Steps use, had lower restrictive practice rates per 1,000 bed days than Cluster 2, which included one-on-ones and more variable Safe Steps use. With the assumption that de-escalation is a form of brief psychotherapeutic intervention, these findings softly challenge the widely-held yet empirically under-explored view that uniform responding may be linked to increased aggression and consequent restrictive practice use (91) and that individual characteristics can moderate psychotherapy outcomes (92). This interpretation is supported by discharged patient interviews from the larger study, where therapeutic connections were said to form even in brief 5–10-minute interactions, when nurses offer genuine, relatable moments. Nurse focus groups from the larger study also revealed how understaffing could have led to random nurse assignments for one-on-one time, which may have been experienced as lacking in relational continuity or even coercive. On the other hand, there is a potential for social desirability bias in log completion, specifically concerning the uniform Safe Steps use in Cluster 1. This bias may be low, given the limited personal benefit from the task expressed by several nurse focus group participants from the larger study. Distortion in self-reports is more commonly observed in high-stakes contexts, such as compensation assessments, where impression management is driven by external incentives (93). Nonetheless, there is a notable absence of comparable tools for detecting ‘faking good’ in de-escalation logs that can support verifying such bias; thus, its presence remains open to question.

### Limitations

There is a limitation in the fidelity checks. None were completed during the first and second time points. However, the de-escalation log completion rates remained high across all the implementation quarters, suggesting that the Safe Steps were actively deployed when fidelity was not checked. Also, excessive fidelity monitoring in reflective practice-based interventions may be counterproductive, as nurses may feel monitored for performance review, which can undermine the psychological safety needed for honest change. In contrast, many successful restrictive practice-reduction interventions were said to depend on giving staff the latitude to exercise their authentic voice (23, 94). This reasoning indicates that making intervention values explicit, rather than imposing compliance, could support staff to move from rewards passivity (where staff receive approval for compliance) towards empowerment, where approval to work towards the agreed values is unnecessary.

The Safe Steps evaluation has further limitations. No demographics were statistically modelled, although such modelling was not pre-specified in the evaluation’s protocol. Since the unit of analysis was at the daily aggregate level, and restrictive practices are often rare and zero-inflated, case-mix adjustment would be fragile without large, balanced subgroups (95), making this demographic modelling omission methodologically justified. Administratively recorded sex was in binary terms, which offers limited clinical relevance in a time of increasing recognition for gender diversity (96). Many administratively recorded mental distress diagnoses were not uniformly classified through standard codes, which limits reliable reporting. Nonetheless, summary data indicated that the sex and diagnostic profiles of individuals were broadly consistent with national patterns among people admitted involuntarily to Australian public acute mental health units (4), supporting the generalisability of the evaluation outcomes to similar service contexts. Nurses’ demographics were excluded to protect anonymity, as required in the obtained ethical approvals. High Australian mental health workforce turnover (97) also complicates longitudinal demographic tracking. Moreover, since mental health nursing is not a protected title in Australia (98), that is, anyone could use the ‘mental health nurse’ designation, regardless of actual educational qualifications or professional competence, a heterogeneous mix of nurse participants likely implemented the Safe Steps, which could reflect typical acute inpatient staffing.

## Conclusion

The Safe Steps evaluation needs to be replicated. The intervention emerged bottom-up, with components co-designed with peer workers, Aboriginal Elders, cultural health leads, and cross-disciplinary professionals. There were a preregistered protocol, *a priori* model-based power analyses, and a sufficiently powered sample, representative of Australian acute mental units. Valid and reliable outcomes, verified through institutional reporting, were tracked over multiple time points, enabling detection of emergent outcomes. Independent cultural and clinical oversight was present. Blinded statistical analyses were undertaken. An implementation and evaluation framework was used, change mechanisms were proposed, nurses’ therapeutic responses were made visible, and a significantly lower as-needed IM psychotropics rate in implementation sites versus controls was found. This lower rate in as-needed IM psychotropic use may reflect a shift toward less coercive practices that reduce trauma and support autonomy, as such interventions are often experienced as disempowering and violent (99). Although IPW was applied and could be considered to have strengthened the plausibility of a causal interpretation, as discussed throughout, chance and some form of biases remain possible. Nonetheless, as the evaluation addresses key evidence gaps, independent replication is needed to strengthen confidence in its findings.

## Data Availability

The datasets presented in this article are not readily available, because of the conditions of the Human Research Ethics Committee approvals obtained. Requests to access the datasets should be directed to human.ethics@scu.edu.au.
