# Supplementary material for: Maximising relational capabilities and minimising restrictive practices in acute mental health units: the Safe Steps for De-escalation evaluation
Source: Front Psychiatry. 2025 Nov 6;16:1676743. doi: 10.3389/fpsyt.2025.1676743 (PMC12630031; doi:10.3389/fpsyt.2025.1676743)
Supplement: Supplementary file 1 [file SupplementaryFile1.docx]

# Supplementary Materials

**Table of Contents**

[Supplementary Materials 1](#_Toc203580093)

[Table 1. Specifications of weighted models 2](#_Toc203580094)

[Figure 2. Love plots 3](#_Toc203580095)

[Figure 3. Silhouette plot 4](#_Toc203580096)

[Table 2. Response fields in the de-escalation log, and brief descriptions of nursing de-escalation practices. 5](#_Toc203580097)

## Table 1. *Specifications of weighted models*

| **Outcomes** | **Between-group Comparison at Baseline** | **Between-group Comparison During Implementation** | **Within-group Comparison** | **Between-Cluster Comparison (not weighted)** |
| --- | --- | --- | --- | --- |
| Total Restrictive Practices | ZIP: RPC ~ CON + (1 \| UNI) | ZIP: RPC ~ CON + (1 \| RDY) | ZIP: RPC ~ TIM + (1 \| UNI) | POISSON: RPC ~ Cluster + (1 \| RDY + MOS) |
| Seclusion | POISSON: SEC ~ CON + (1 \| RDY) | POISSON: SEC ~ CON + (1 \| RDY) | POISSON: SEC ~ TIM + (1 \| RDY) | ZIP: SEC ~ Cluster + (1 \| MOS) |
| Physical Restraint | ZIP: RES ~ CON + (1 \| RDY) | ZIP: RES ~ CON + (1 \| RDY) | POISSON: RES ~ TIM + (1 \| RDY) | ZIP: RES ~ Cluster + (1 \| NOS) |
| Intramuscular Psychotropic use | ZIP: SED ~ CON + (1 \| UNI) | ZIP: SED ~ CON + (1 \| RDY) | ZIP: SED ~ TIM + (1 \| UNI) | POISSON: SED ~ Cluster + (1 \| RDY + MOS) |
| Physical Injury | ZIP: TPI ~ CON + (1 \| UNI) | ZIP: TPI ~ CON + (1 \| MOS) | ZINB: TPI ~ TIM + (1 \| DAY) | ZIP: TPI ~ Cluster + (1 \| NOS) |
| Code Black | ZIP: CDB ~ CON + (1 \| RDY) | POISSON: CDB ~ CON + (1 \| RDY) | ZIP: CDB ~ TIM + (1 \| UNI) | Did not converge |
| Total Restrictive Practice Duration | LMM: RPD ~ CON + (1 \| UNI + DAY + YER) | LMM: RPD ~ CON + (1 \| UNI + YER + RDY) | LMM: RPD ~ TIM + (1 \| UNI + RDY) | LM: RPD ~ Cluster |
| Seclusion Duration | LMM: SECD ~ CON + (1 \| DAY + YER) | LMM: SECD ~ CON + (1 \| UNI + YER + RDY) | LMM: SECD ~ TIM + (1 \| UNI + RDY) | LM: SECD ~ Cluster |
| Physical Restraint Duration | LMM: RESD ~ CON + (1 \| UNI) | LMM: RESD ~ CON + (1 \| UNI + MOS + RDY) | LMM: RESD ~ TIM + (1 \| UNI + DAY + MOS) | LM: RESD ~ Cluster |

*Note.*. Model type codes: ZIP = zero-inflated Poisson regression; Poisson = standard Poisson generalised linear mixed model; LMM = linear mixed model for continuous outcomes. Fixed effect: CON = between-group independent variable (i.e., implementation or control condition). Random effects: specified using (1 | GROUP), where | denotes nesting. Covariate codes: UNI = unit; RDY = study day index; DAY = day; MOS = month; YER = year. Outcome codes: RPC = total restrictive practice event; SEC = seclusion events; RES = physical restraint events; SED = intramuscular pharmacological response; TPI = total physical injuries; CDB = Code Black activations; RPD = total restrictive practice duration; SECD = seclusion duration; RESD = physical restraint duration.

## Figure 2. Love plots


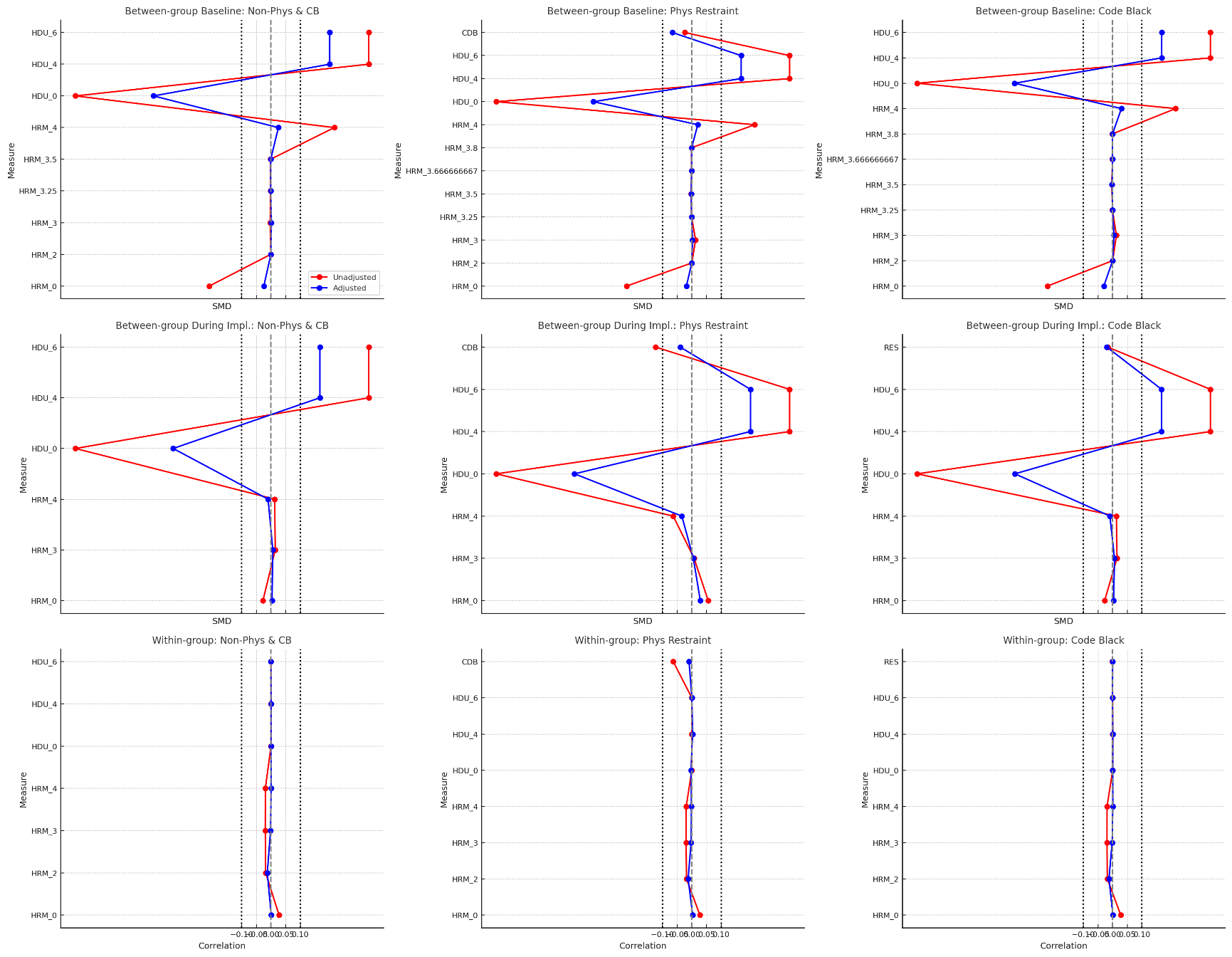


## Figure 3. Silhouette plot


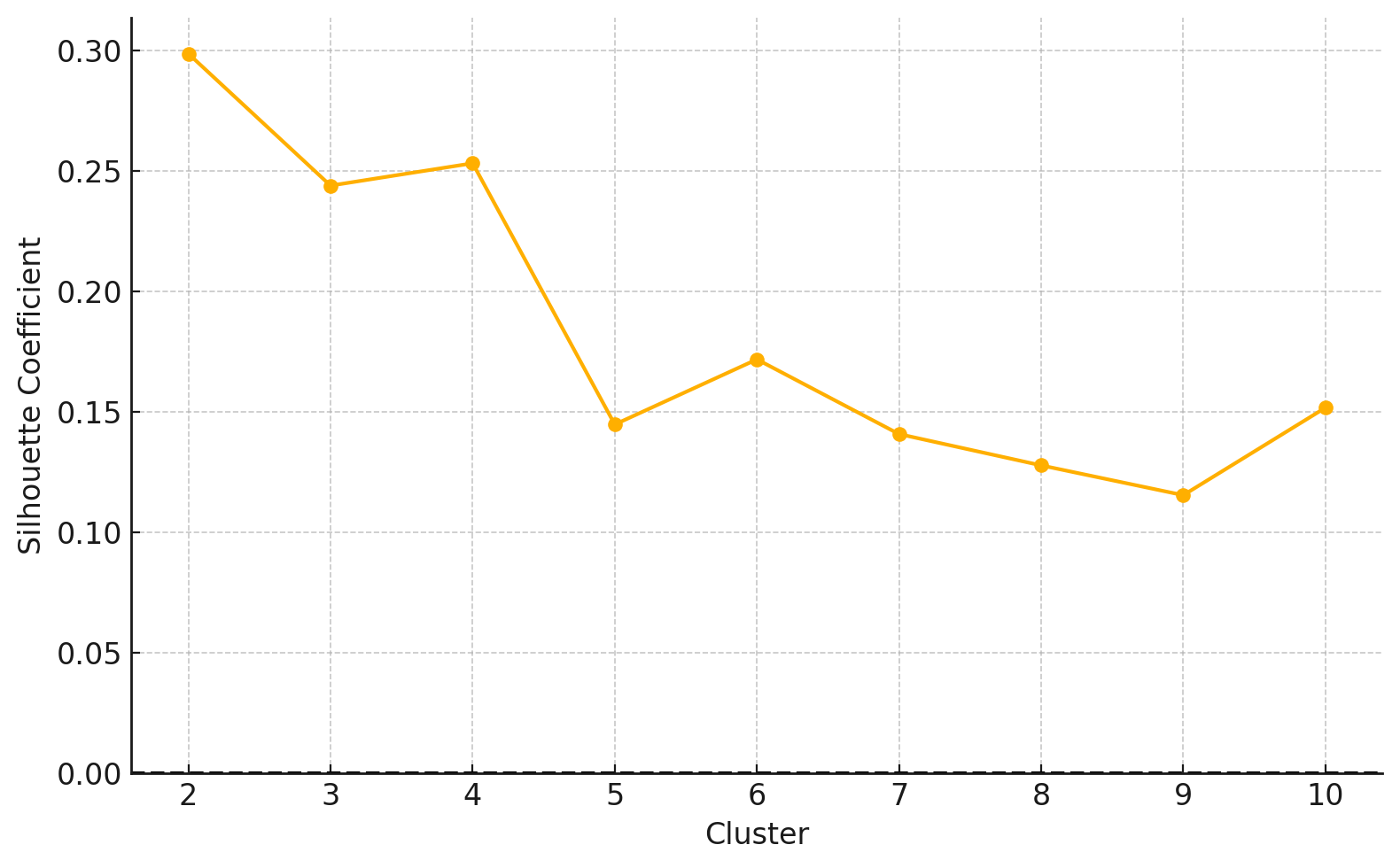


## Table 2. Response fields in the de-escalation log, and brief descriptions of nursing de-escalation practices.

| **Event Code** | **Description** |
| --- | --- |
| AM | Incident occurred during the morning shift |
| PM | Incident occurred during the afternoon shift |
| Night | Incident occurred during the night shift |
| HOA | Incident occurred in a high observation area |
| LOA | Incident occurred in a low observation area |
| NOS | Number of nurses involved in de-escalation |
| DCA | Person was calm or only agitated (not aggressive) |
| DAP | Aggression directed toward another person receiving care |
| DSH | Self-harming behavior |
| DPS | Aggression directed toward nurses or unit staff |
| DPV | Aggression directed toward a visitor, by the person |
| DVP | Aggression directed toward the person, by a visitor |
| DVS | Aggression directed toward staff, by a visitor |
| DPH | Aggression directed toward property or inanimate objects |
| SS1 | Staff emotional self-regulation or self-management: Nurses adjust their own emotional state during interactions. This may involve changes in tone, breathing, or posture to convey calmness. |
| SS2 | Identify the issue or problem: Nurses help the person articulate, recognise, or reframe the source of distress. Techniques include active listening, rapport-building, and clarifying triggers. |
| SS3 | Acknowledge and validate: Nurses reflect or name the person's emotional or cognitive state. This includes verbal affirmations, emotional labelling, or refocusing discussion. |
| SS4 | Work toward agreeable solution: Nurses and the person jointly explore next steps. This may involve negotiating options and reaching shared understanding. |
| SS5 | Establish expected behaviour: Nurses set clear expectations or boundaries. This may include reminders about roles, safety policies, or unit rules. |
| IDI | Distraction: Nurses introduce alternative activities or topics to redirect attention. Techniques include humour, casual conversation, or tactile objects. |
| IRE | Redirection: Nurses guide the person toward a different purpose using verbal prompts or non-verbal cues to interrupt escalation. |
| ICE | Change environment: Nurses accompany or direct the person to a different area. The new space may be quieter or more regulated. |
| IRS | Reduced stimulus: Nurses lower sensory inputs (e.g., noise, light, presence of others). Actions include dimming lights or reducing room occupancy. |
| IMU | Music: Nurses provide access to music via headphones, speakers, or devices. Music may be person-selected or unit-provided. |
| IOP | Oral PRN (pro re nata; as-needed) medication: Nurses administer prescribed medication based on clinical assessment and policy. |
| IIN | Individualised staff time: Nurses spend time with the person outside routine care. Activities may include sitting together, talking, or assisting with hygiene. |
| IFD | Food and drinks: Nurses provide snacks or beverages, either routinely or in response to agitation. |
| ICS | Culturally sensitive care: Nurses support cultural or spiritual practices (e.g., prayer space, traditional foods, identity-affirming items). |
| ISM | Sensory modulation: Nurses provide sensory tools (e.g., weighted blankets, textured items, aromatherapy). |
| IPC | Phone call: Nurses assist the person to make or receive phone calls. These may be supervised or unsupervised and involve personal or official matters. |
| PRD | Immediacy of even prior to de-escalation, rated on a 6-point scale: i) calm, ii) agitation, iii) verbal aggression, iv) physical aggression to properties, v) physical aggression towards people or self, vi) physical aggression with weapons. |
